# The Association Between an Addictive Tendency Toward Food and Metabolic Characteristics in the General Newfoundland Population

**DOI:** 10.3389/fendo.2018.00661

**Published:** 2018-11-09

**Authors:** Matthew Nelder, Farrell Cahill, Hongwei Zhang, Guangju Zhai, Wayne Gulliver, Weiping Teng, Zhongyan Shan, Guang Sun

**Affiliations:** ^1^Complex Disease Laboratory, Faculty of Medicine, Memorial University of Newfoundland, St. John's, NL, Canada; ^2^Liaoning Provincial Key Laboratory of Endocrine Diseases, Department of Endocrinology and Metabolism, Endocrine Institute, First Hospital of China Medical University, Shenyang, China

**Keywords:** food addiction, yale food addiction scale, obesity, insulin resistance, lipids

## Abstract

**Background:** Our previous study of 29 obese food addiction (FA) patients found that FA is associated with lipid profiles and hormones which may be a factor in cardiovascular disease (CVD) and insulin resistance (IR). However, there is currently no data available regarding the relationship between FA symptoms and metabolic characteristics of CVD and IR in the general population. We designed this study to investigate the correlation between FA symptoms with lipid profiles and IR in men and women of the general Newfoundland population.

**Methods:** 710 individuals (435 women and 275 men) recruited from the general Newfoundland population were used in analysis. FA symptoms were evaluated using the Yale Food Addiction Scale (YFAS). Glucose, insulin, HDL, LDL, total cholesterol and triglycerides levels were measured. IR was evaluated using the homeostatic model of assessment (HOMA). Participants were grouped by sex and menopausal status. Age, physical activity, calories and total % body fat were controlled.

**Results:** Partial correlation analysis revealed that in men, YFAS symptom counts were significantly correlated with HOMA-β (*r* = 0.196, *p* = 0.021), triglycerides (*r* = 0.140, *p* = 0.025) and inversely correlated with HDL (*r* = −0.133, *p* = 0.033). After separating by menopausal status, pre-menopausal women exhibited no correlations and post-menopausal women had a significantcorrelation with triglycerides (*r* = 0.198, *p* = 0.016).

**Conclusion:** FA is significantly correlated with several markers of metabolic disturbance in men and to a lesser extent, post-menopausal women, in the general population. Further research is required to explain sex specific associations and elucidate any potentially causal mechanisms behind this correlation.

## Introduction

Obesity rates have been climbing for the last three decades and according to a report from Statistics Canada, in Newfoundland (NL), 31% of adult women and 27.3% of men are obese (1, 2). It follows that the population of NL also exhibit some of the highest rates of obesity related health issues in Canada such as type II diabetes (TIID) and cardiovascular disease (CVD) which makes obesity research within this population so vital. Recent reports put the rate of TIID in this Province at 11.2% with CVD contributing to 20–38% of total deaths in adults aged 35 years or older (3–5). The development of obesity is highly complex and is comprised of a number of interacting genetic, environmental and behavioral factors (6–9). One issue that highlights this intricate balance of factors is that of an addictive tendency toward food. This is characterized by an obsessive consumption of foods known to be highly palatable with corresponding activation of the bodies reward systems and significant difficulty controlling this behavior (10). Previous research on food addiction discovered there are tangible neural correlates in the way of dopaminergic circuitry activity and it has drawn comparisons to nicotine addiction in regards to how the body's reward systems are effected (11, 12).

Symptoms of an addictive tendency toward food are quantified using the Yale Food Addiction Scale (YFAS) which has been adapted from the criteria for substance abuse from the DSM-IV TR (13). Foods that display addictive qualities are those that are high in simple sugars, saturated fat and are heavily processed. One study revealed that the processing of food was a strong predictor of it possessing addictive qualities (14). There is substantial evidence that these very same types of foods can have a significant negative impact on health. Foods high in fructose can be highly lipogenic and could lead to an increased risk of developing non-alcoholic fatty liver disease and consequently, TIID (15). A study concerning consumption of sugar sweetened beverages effects on health looked at over 310,000 individuals and over 15,000 TIID patients and found that those in the highest quartile of sugar sweetened beverage consumption had a 26% higher risk of developing TIID (16). Excessive sucrose intake has also been found to be associated with a 37% increased risk of having a coronary event (17). Furthermore, a 13 years follow up study performed on 4,999 individuals found that consumption of milk fats, various sweets and cake was associated with increased CVD risk in women (18). In addition to the behavioral and neurological evidence supporting the role of addictive food tendencies in health, we have recently found that there are different hormonal characteristics between food addicted (FA) and non-food addicted (NFA) individuals.

We have previously discovered that obese food addicts had lower levels of thyroid stimulating hormone (TSH), tumor necrotic factor alpha (TNF-α) and amylin but higher levels of prolactin compared to non-food addicted obese individuals (19). TSH levels have previously been implicated in the development of insulin resistance (IR) and diabetes while TNF-α has been shown to decrease appetite, therefore, lower levels of TNF-α may exasperate obesity related comorbidities by lending to increased positive energy imbalance (20–22). Amylin is known to be associated with development of type 1 and TIID and increased prolactin levels have been shown to be a cardiovascular risk factor with correlations to diabetes as well (23–26). However, our previous study was performed with only 58 individuals (29 FA obese, 29 NFA obese). Many studies in the field of “food addiction” focus on specific sub-populations such as the obese, bariatric surgery patients or those with an eating disorder (27, 28). What remains to be seen is whether or not an addictive tendency toward food displays an association with metabolic markers and hormones in the general population, specifically IR and lipid profiles as these metabolic characteristics play a role in the development of TIID and CVD (29, 30).

The very same types of foods that possess addictive qualities are demonstrably dangerous to an individual's overall health when frequently consumed. It is therefore essential that the association between addictive tendencies toward food and metabolic markers of TIID and CVD be studied in the general population. Our investigation was performed in a sub-group of the CODING (Complex Diseases in a Newfoundland Population: Environment and Genetics) study which consists of biochemical and body composition data from over 3,200 men and women from the general Newfoundland population.

## Materials and methods

### Participants

Seven hundred and ten adults (435 women, 275 men) from the Province of Newfoundland and Labrador were recruited for this study through the use of advertisements, flyers and word of mouth. Eligibility requirements are as follows: (1) 19 years of age or older. (2) The participant must have been born in NL and the participant's family must have been in NL for at least 3 generations. (3) Healthy with no serious metabolic, endocrine or cardiovascular conditions. (4) Participant must not be pregnant at time of study.

We have also excluded participants who have been found to have “food addiction” in our previous study, from correlational analysis but later included food addicted individuals for an additional comparative analysis. Individuals who self-reported having diabetes or hypertension were also excluded from this analysis.

### Food addiction symptom count measurement

Symptoms of food addiction were assessed using the YFAS. This is a 25-item questionnaire which assesses the last 12 months of eating patterns. The YFAS is based on the criteria used for substance dependence in the Diagnostic and Statistical Manual IV TR (DSM-IV TR). This criterion includes symptoms such as tolerance, withdrawals, anxiety in social situations, and difficulty lowering or quitting use of substance. Symptoms were counted using the Likert scoring method which ranges from 0 to 7 (13). Each symptom on the YFAS represents a collection of questions concerning that particular behavior. If a participant answers affirmatively to certain questions, they are said to have the corresponding symptom. Some of these questions are based on the frequency of the behavior (ex. once a month, 2–4 times a month, etc.) and some are binary yes or no questions (10). In our comparison of food addicts and non-food addicts, those classified as food addicts were required to have at least 3 symptoms and answer affirmatively to one of the two questions from the YFAS which indicate clinically significant dependence.

### Caloric intake and physical activity assessment

Calorie intake was measured using the Willett Food Frequency Questionnaire (FFQ) which measures the last 12 months of dietary habits (31). The quantity of each food item was converted into a daily average intake value. This value was then entered in the Nutribase Clinical Nutrition Manager (software version 9.0; Cybersoft inc. Arizona). We employed the use of the Baecke physical activity questionnaire to assess physical activity levels. This questionnaire measures physical activity using three categories which are work, sport and leisure.

### Body composition and metabolic marker measurements

Body composition measurements, which in this study included total body fat, was assessed using dual-energy x-ray absorptiometry (DXA; Lunar Prodigy; GE Medical Systems, Madison, WI, USA). Measurements were taken while patient lied horizontally on DXA scanner following a 12 hour fast. Serum concentrations of glucose, total cholesterol, HDL cholesterol and triacylglycerols were measured using the Lx20 analyzer (Beckman Coulter Inc., Fullerton, CA) with Synchron reagents. LDL cholesterol was calculated using the following formula: (total cholesterol)—(HDL cholesterol)—(triacylglycerols/2.2). Serum insulin was measured using the Immulite Immunoassay analyzer. Insulin resistance and beta cell function were calculated using the homeostasis model assessment (HOMA) (32).

### Statistical analysis

All statistical analysis was completed using SPSS ver.23. The level of statistical significance was set at *P* < 0.05. Statistical tests were run on men and women separately. Pearson correlation analysis was used to evaluate potential relationships between YFAS symptom counts and all metabolic markers. Partial correlation analysis was also performed which allowed us to control for age, total calorie intake, physical activity levels, and total body fat % as necessary. An independent *t*-test was run between BMI matched food addicts and a control group to assess the difference of mean in the metabolic markers that were measured. Control variables were selected for each gender dependent on whether the potentially confounding variable in question shared a significant association with both dependent and independent variables. Following this method, we chose to control for age, physical activity, total body fat % and calories. In order to better normalize distribution of the data, HOMA-IR, and HOMA-β were square root transformed.

## Results

### Physical characteristics and adiposity measures

The age, gender, BMI and anthropometric data of all men and women who participated in this study are presented in Table 1A as mean ± standard deviation. The average age of female participants was 45.4 years and the average age of male participants was 42.9 years. There was a significant difference observed between men and women for height, weight, BMI, total % body fat, waist and hip measurements with men being higher for each measure. There was also a significant difference observed between pre and post-menopausal women for age, height, waist and hip circumference as well total body fat % which is highlighted in Table 1B.

**Table 1A:** Physical characteristics of participants.

	**Female**	**Male**
	**Mean ±*SD***	**Mean ±*SD***
	***n* = 435**	***n* = 275**
Age^*^	45.4 ± 13.4	42.9 ± 12.9
Min-max	18.1–90.0	18.9–78.5
Height (cm)^*^	163.3 ± 6.1	177.6 ± 6.6
Min-max	144.0–187.6	156.8–200.0
Weight (kg)^*^	70.1 ± 14.6	90.5 ± 17.2
Min-max	44.2–132.2	57.1–149.5
BMI^*^	26.3 ± 5.5	28.3 ± 4.6
Min-max	16.9–53.6	19.0–43.3
BF%^*^	36.6 ± 8.6	26.2 ± 8.3
Min-max	12.5–58.6	5.3–45.5
Waist (cm)^*^	90.4 ± 13.5	100.9 ± 13.4
Min-max	62.0–139.5	64.9–140.0
Hip (cm)^*^	99.1 ± 12.6	101.4 ± 10.6
Min-max	75.0–148.0	78.0–135.0
Waist/Hip (cm)^*^	0.91 ± 0.07	1.0 ± 0.05
Min-max	0.68–1.62	0.75–1.14

* *significant difference between men and women (p < 0.05) Mean ± Standard Deviation (SD), (Minimum-Maximum), BF%- Total Body Fat Percent*.

**Table 1B:** Physical characteristics of pre and post-menopausal women.

	**Pre**	**Post**
	**Mean ±*SD***	**Mean ±*SD***
	***n* = 277**	***n* = 158**
Age^*^	37.7 ± 9.3	58.2 ± 8.3
Min-max	19-56	37-90
Height (cm)^*^	164.2 ± 6.16	161.8 ± 5.66
Min-max	144–188	150.0–179.0
Weight (kg)	69.2 ± 14.3	71.6 ± 15.0
Min-max	39.0–119.0	48.0–132.0
BMI	25.7 ± 5.32	27.2 ± 5.53
Min-max	17.0–44.4	18.2–54.0
BF%^*^	34.9 ± 8.91	39.4 ± 7.40
Min-max	13.0–56.0	20.0–58.6
Waist (cm)^*^	88.0 ± 13.4	94.2 ± 12.8
Min-max	63.0–139.5	62.0–139.0
Hip (cm)^*^	96.9 ± 12.4	102.6 ± 12.0
Min-max	74.0–140.0	75.0–148.0
Waist/Hip (cm)	0.91 ± 0.08	0.92 ± 0.06
Min-max	0.75–1.62	0.68–1.06

* *significant difference between men and women (p < 0.05)Mean ± Standard Deviation (SD), (Minimum-Maximum), BF%- Total Body Fat Percent*.

### Metabolic characteristics

Men had much higher levels of glucose and triglycerides compared to women. Women, however, had significantly higher levels of HDL cholesterol (Table 2A). All other markers including insulin, HOMA-IR, HOMA-β, LDL and total cholesterol were not significantly different between men and women. Metabolic characteristics were also compared between pre and post-menopausal women (Table 2B). Post-menopausal women had notably higher levels of insulin, glucose, and triglycerides in addition to higher levels of both LDL and total cholesterol.

**Table 2A:** Metabolic characteristics of men and women.

	**Female**	**Male**
	**Mean ±*SD***	**Mean ±*SD***
	***n* = 435**	***n* = 275**
Insulin	77.8 ± 94.1	79.3 ± 53.7
HOMA-IR	2.90 ± 5.50	3.20 ± 3.30
HOMA-β	136.9 ± 123.8	122.8 ± 78.1
Glucose^*^	5.06 ± 0.60	5.55 ± 1.52
Triglycerides^*^	1.09 ± 0.69	1.35 ± 1.00
HDL^*^	1.51 ± 0.37	1.23 ± 0.29
LDL	2.98 ± 0.89	3.13 ± 1.00
Cholesterol	5.05 ± 0.96	5.06 ± 1.08

* *significant difference between men and women (p < 0.05)Mean ± Standard Deviation (SD), HOMA-IR, Homeostatic Model of Assessment-Insulin Resistance; HOMA-β, Homeostatic Model of Assessment-Beta Cell; HDL, High Density Lipoprotein; LDL, Low Density Lipoprotein*.

**Table 2B:** Metabolic characteristics of pre and post menopausal women.

	**Pre**	**Post**
	**Mean ±*SD***	**Mean ±*SD***
	***n* = 277**	***n* = 158**
Insulin^*^	66.0 ± 50.6	92.4 ± 127.9
HOMA-IR	2.23 ± 1.62	3.68 ± 7.86
HOMA-β	139.4 ± 139.1	134.8 ± 104.2
Glucose^*^	4.93 ± 0.46	5.28 ± 0.74
Triglycerides^*^	0.98 ± 0.61	1.27 ± 0.77
HDL	1.52 ± 0.36	1.51 ± 0.38
LDL^*^	2.74 ± 0.78	3.38 ± 0.90
Cholesterol^*^	4.80 ± 0.83	5.51 ± 0.99

* *significant difference between pre and post-menopausal women (p < 0.05) Mean ± Standard Deviation (SD), HOMA-IR, Homeostatic Model of Assessment-Insulin Resistance; HOMA-β, Homeostatic Model of Assessment-Beta Cell; HDL, High Density Lipoprotein; LDL, Low Density Lipoprotein*.

### Correlations between YFAS symptoms and metabolic markers

The Pearson and partial correlation coefficients as well as *p*-values are presented in Tables 3–6. Men exhibited significant positive correlations between YFAS symptoms and HOMA-β, TG as well as a significant negative correlation with HDL cholesterol (Table 3). In men the correlation between YFAS symptoms and HOMA-IR just barely missed the cut-off of for statistical significance at α = 0.05 (*r* = 0.164, *p* = 0.053). These correlations remained significant after controlling for age, physical activity, total body fat % and calories. YFAS symptoms counts in women exhibited nosignificant correlations (Table 4) however, when separated by menopausal status, post-menopausal (Table 5) women showed a significant correlation with TG (*r* = 0.198) after controlling for age, physical activity, total body fat % and calories. Pre-menopausal women exhibited no significant correlations after controlling for all confounding variables (Table 6). The correlation between YFAS symptoms and HOMA-β was strongest for men and TG exhibited the only significant correlation strongest for post-menopausal women.

**Table 3:** Correlations between YFAS symptom counts and metabolic characteristics in men.

**YFAS Symptom Counts**	**Men (*****n*** = **275)**				
	**r1(P)**	***r*****2(P)**	***r*****3(P)**	***r4(P)***	***r5(P)***
Insulin (pmol/L)	0.248 (0.001)^*^	0.248 (0.001)^*^	0.231 (0.002)^*^	0.140 (0.064)^*^	0.135 (0.080)
HOMA-IR	0.262 (0.001)^*^	0.271 (0.001)^*^	0.255 (0.002)^*^	0.167 (0.048)^*^	0.164 (0.053)
HOMA-β	0.316 (0.000)^*^	0.302 (0.000)^*^	0.283 (0.001)^*^	0.201 (0.016)^*^	0.196 (0.021)^*^
Glucose (mmol/L)	−0.075 (0.217)	−0.040 (0.511)	−0.048 (0.440)	−0.075 (0.224)	−0.070 (0.260)
TG (mmol/L)	0.205 (0.001)^*^	0.222 (0.000)^*^	0.203 (0.001)^*^	0.137 (0.026)^*^	0.140 (0.025)^*^
HDL (mmol/L)	−0.232 (0.000)^*^	−0.222 (0.000)^*^	−0.201 (0.001)^*^	−0.137 (0.025)^*^	−0.133 (0.033)^*^
LDL (mmol/L)	−0.031 (0.614)	0.004 (0.947)	−0.005 (0.935)	−0.059 (0.342)	−0.055 (0.378)
Cholesterol (mmol/L)	−0.006 (0.917)	0.040 (0.511)	0.020 (0.640)	−0.025 (0.683)	−0.019 (0.756)

* *p-value < 0.05, r1-pearson correlation, r2-controlled for age, r3-controlled for age, physical activity, r4-controlled for age, physical activity, total % body fat, r5-controlled for age, physical activity, total % body fat, calories*.

**Table 4:** Correlations between YFAS symptom counts and metabolic characteristics in women.

**YFAS Symptom Counts**	**Women (*****n*** = **435)**				
	**r1(P)**	***r*****2(P)**	***r*****3(P)**	***r4(P)***	***r5(P)***
Insulin (pmol/L)	0.084 (0.221)	0.086(0.209)	0.069 (0.319)	0.011 (0.880)	0.021 (0.766)
HOMA-IR	0.066 (0.355)	0.064 (0.370)	0.054 (0.458)	0.049 (0.496)	0.036 (0.624)
HOMA-β	0.071 (0.319)	0.064 (0.372)	0.052 (0.468)	0.005 (0.947)	0.021 (0.777)
Glucose (mmol/L)	−0.039 (0.423)	−0.024 (0.622)	−0.042 (0.388)	−0.080 (0.102)	−0.085 (0.081)
TG (mmol/L)	0.122 (0.011)^*^	0.131 (0.007)^*^	0.110 (0.023)^*^	0.059 (0.224)	0.065 (0.152)
HDL (mmol/L)	−0.112 (0.020)^*^	−0.110 (0.022)^*^	−0.082 (0.092)	−0.010 (0.841)	−0.010 (0.835)
LDL (mmol/L)	−0.015 (0.750)	0.004 (0.940)	−0.001 (0.980)	−0.028 (0.567)	−0.030 (0.537)
Cholesterol (mmol/L)	0.011 (0.824)	0.035 (0.465)	−0.032 (0.514)	0.018 (0.714)	0.017 (0.732)

* *p-value < 0.05, r1-pearson correlation, r2-controlled for age, r3-controlled for age, physical activity, r4- controlled for age, physical activity and total % body fat, r5- controlled for age, physical activity and total % body fat, calories*.

**Table 5:** Correlations between YFAS symptom counts and metabolic characteristics in post-menopausal women.

**YFAS Symptom Counts**	**Females (*****n*** = **158)**				
	**r1(P)**	**r2(P)**	**r3(P)**	**r4(P)**	**r5(P)**
Insulin (pmol/L)	0.021 (0.841)	0.013 (0.913)	0.020 (0.853)	−0.027 (0.803)	0.009 (0.936)
HOMA-IR	0.015 (0.889)	0.014 (0.899)	0.018 (870)	−0.024 (833)	0.015 (0.897)
HOMA-β	0.032 (0.763)	0.016 (0.880)	0.033 (0.763)	−0.009 (0.934)	0.032 (0.778)
Glucose (mmol/L)	−0.047 (0.557)	−0.035 (0.664)	−0.058 (0.477)	−0.087 (0.920)	−0.089 (0.223)
TG (mmol/L)	0.227 (0.004)^*^	0.226 (0.004)^*^	0.216 (0.007)^*^	0.191 (0.019)^*^	0.198 (0.016)^*^
HDL (mmol/L)	−0.029 (0.715)	−0.010 (0.902)	0.008 (0.922)	0.064 (0.438)	−0.032 (0.701)
LDL (mmol/L)	−0.098 (0.218)	−0.086 (0.285)	−0.089 (0.274)	−0.084 (0.303)	−0.098 (0.235)
Cholesterol (mmol/L)	0.024 (0.765)	0.045 (0.577)	0.040 (0.626)	0.059 (0.475)	0.041 (0.619)

* *p-value < 0.05, r1-pearson correlation, r2-controlled for age, r3-controlled for age, physical activity, r4-controlled for age, physical activity, total % body fat, r5-controlled for age, physical activity, total % body fat, calories*.

**Table 6:** Correlations between YFAS symptom counts and metabolic characteristics in pre-menopausal women.

**YFAS Symptom Counts**	**Females (*****n*** = **277)**				
	**r1(P)**	**r2(P)**	**r3(P)**	**r4(P)**	**r5(P)**
Insulin (pmol/L0	0.141 (0.120)	0.142 (0.119)	0.113 (0.227)	0.052 (0.571)	0.053 (0.572)
HOMA-IR	0.087 (0.366)	0.087 (0.369)	0.071 (0.468)	0.073 (0.455)	0.059 (0.546)
HOMA-β	0.097 (0.314)	0.103 (0.285)	0.076 (0.432)	0.031 (0.753)	0.037 (0.709)
Glucose (mmol/L)	−0.012 (0.843)	−0.017 (0.784)	−0.032 (0.602)	−0.073 (0.234)	−0.079 (0.201)
TG (mmol/L)	0.078 (0.200)	0.077 (0.204)	0.049 (0.418)	−0.016 (0.790)	−0.012 (0.842)
HDL (mmol/L)	−0.162 (0.007)^*^	−0.163 (0.007)^*^	−0.128 (0.035)^*^	−0.054 (0.377)	−0.047 (0.441)
LDL (mmol/L)	0.066 (0.277)	0.064 (0.288)	0.066 (0.282)	0.017 (0.778)	0.017 (0.788)
Cholesterol (mmol/L)	0.038 (0.528)	0.036 (0.556)	0.036 (0.559)	−0.001 (0.985)	0.001 (0.990)

* *p-value < 0.05, r1-pearson correlation, r2-controlled for age, r3-controlled for age, physical activity, r4-controlled for age, physical activity, total % body fatr5-controlled for age, physical activity, total % body fat, calories*.

### Comparison of food addicts and non-food addicts

No significant difference was observed between BMI matched FA and NFA individuals from the general population for markers of IR or lipid profiles. These groups consisted of obese, overweight and normal weight individuals. When separated by sex, there was still no statistically significant differences observed in men or women (Table 7). FA men had significantly lower HDL levels than FA women.

**Table 7:** Metabolic Marker Measures in BMI Matched Food and Non-Food Addicted Individuals.

	**Total Population**		**Men**		**Women**	
	**FA**	**NFA**	**FA**	**NFA**	**FA**	**NFA**
	**Mean** ±***SD***	**Mean** ±***SD***	**Mean** ±***SD***	**Mean** ±***SD***	**Mean** ±***SD***	**Mean** ±***SD***
	***n*** = **38**	***n*** = **38**	***n*** = **9**	***n*** = **9**	***n*** = **29**	***n*** = **29**
Insulin (pmol/L)	122.1 ± 132.2	87.9 ± 41.8	231.3 ± 84.6	96.4 ± 33.9	81.9 ± 35.4	84.4 ± 45.0
HOMA-IR	0.75 ± 1.40	0.45 ± 0.20	1.99 ± 2.85	0.50 ± 0.15	0.40 ± 0.22	0.43 ± 0.22
HOMA-β	2.14 ± 0.24	2.13 ± 0.17	2.20 ± 0.40	2.15 ± 0.18	2.12 ± 0.18	2.12 ± 0.18
Glucose (mmol/L)	5.44 ± 1.20	5.14 ± 0.41	6.37 ± 2.09	5.29 ± 0.36	5.14 ± 0.50	5.09 ± 0.42
TG (mmol/L)	1.32 ± 0.70	1.29 ± 0.75	1.50 ± 0.69	1.50 ± 0.95	1.26 ± 0.71	1.23 ± 0.68
HDL (mmol/L)^*^	1.36 ± 0.35	1.37 ± 0.31	1.06 ± 0.17	1.12 ± 0.26	1.46 ± 0.34	1.44 ± 0.29
LDL (mmol/L)	3.18 ± 1.07	3.00 ± 0.77	3.06 ± 1.48	3.09 ± 0.45	3.22 ± 0.93	2.97 ± 0.85
Cholesterol (mmol/L)	5.19 ± 1.23	5.00 ± 0.91	4.80 ± 1.52	4.88 ± 0.74	5.32 ± 1.14	5.04 ± 0.97

* *significant difference between FA men and women at p < 0.05 Mean ± standard deviation(SD) FA, Food Addict; NFA, Non-Food Addict; HOMA-IR, Homeostatic Model of Assessment-Insulin Resistance; HOMA-β, Homeostatic Model of Assessment-Beta Cell; TG, Triglyceride; HDL, High Density Lipoprotein; LDL, Low density Lipoprotein*.

## Discussion

In Canada, between 1985 and 2011 the rate of obesity leapt from 6.1 to 18.3% (33). Not surprisingly, the rapid rise in obesity over the same time span is paralleled by a substantial rise in diabetes both in Canada and globally (34, 35). There is evidence supporting the role of processed and fast foods as key contributors to the obesity epidemic and it is worth noting that these same types of foods may override the body's normal satiety signals leading to overconsumption (36–40). Similar findings have been produced in animal studies. Rats who were subjected to stress consumed up to 160% more food when given just a taste of highly palatable food as opposed to rats given just standard chow (41). While hunger related hormones such as ghrelin and leptin were not included in our analysis, the role they potentially play in food addiction and the metabolic consequences thereof, should not be understated. Leptin, one of the bodies main satiety promoting hormones, was found to decrease feeding-evoked dopamine release in rats while another study found that ghrelin signaling, which induces hunger, is key to driving the consumption of “rewarding” foods. This study also showed that dopamine release was absent in ghrelin receptor knock-out mice (42, 43). This is important to consider from a metabolic health standpoint as well because other animal studies have also shown that leptin may reduce adiposity independent of caloric intake which suggest leptin levels could also affect other metabolic characteristics, even if indirectly (44). Furthermore, an investigation of the effects of a ghrelin infusion found that glucose utilization in rats increased in both white and brown fat cells (45). It is possible that the levels of these hunger related hormones in our own cohort may have had some effect on the metabolic characteristics that were measured. The very same foods which possess addictive qualities, can lead to a cascade of metabolic disturbances if frequently consumed (16, 17, 46, 47) which contribute to the development of diabetes and CVD (48–50). This marks the need for more exploration of the relationship between an addictive tendency toward food and health. No study, to our knowledge, has explored the association between an addictive tendency toward food and metabolic characteristics in the general population. A better understanding of how symptoms of food addiction are associated with IR and lipid profiles would help us better comprehend the impact of specific eating behaviors on health.

The main finding produced from our study was that in adult males, despite not meeting the criteria for a “food addiction” diagnosis, YFAS symptom counts were still significantly, associated with HOMA-β and TG levels as well as inversely associated with HDL levels. Both HOMA-IR and HOMA-β have been implicated in diabetes risk in the past (51). HOMA-β, a measure of insulin secretion, has also been found to initially parallel rising TG levels in newly diagnosed type 2 diabetics only to decrease as TG levels continued to rise higher (52). Interestingly, HOMA-β levels tend to be lower in individuals with a family history of diabetes due to lower levels of insulin secretion as a result of impaired β-cell function (53). The aforementioned studies demonstrate the complexity of the relationship between insulin secretion and metabolic health. In the current study, there was a significant positive correlation between HOMA-β and YFAS symptom counts which may suggest that food addicted behaviors result in higher insulin secretion as a compensatory mechanism. This could potentially be problematic as consistently high insulin levels can often precede diabetes which can eventually damage the β-cell to the point where the cell can no longer produce sufficient levels of insulin to combat hyperglycemia. This is evidenced by the enlargement of β-cells observed in obese individuals and those in the pre-diabetic phase (54–56). HOMA-β measurements, therefore, must be interpreted cautiously as high insulin secretion can suggest an individual is at risk of developing diabetes and low insulin secretion may suggest that the damage has already been done to the β-cell. A study by the Canadian Heart Health Surveys Research Group found that in a sample of over 29 000 Canadians, high TG and low HDL levels were common among diabetics and those with hypertension (57). Elevated TG levels have also been associated with atherosclerotic CVD and all cause mortality in a study comprised of over 15 000 patients (58, 59). It stands that behaviors that may influence TG and HDL levels may also increase the risk of CVD. There is currently limited research on the relationship between food addiction, its symptoms and their relationship with these metabolic characteristics, however, one study did find that food addiction was significantly more prevalent in newly diagnosed type 2 diabetics when compared to a control group. This same study also compared the metabolic characteristics of FA and NFA diabetics and found that those who were food addicted had a substantially higher HOMA-IR value (60). However, our own results indicated that there was no significant difference in HOMA-IR or other metabolic characteristics between FA and NFA individuals when matched for BMI. Food addiction has also been found to account for a large portion of BMI variance among T2D patients (61). This may suggest that an addictive tendency toward food is not only a risk factor for T2D but could in fact exasperate the condition since patients will have greater difficulties adhering to dietary recommendations that may improve their health and help manage their condition. As previously noted, some of the hormones associated with addictive behaviors also share associations with a number of health risks which may further compound the effects of an addictive tendency toward food.

In females, Pearson correlation analysis revealed no significant correlations between YFAS symptoms and any of the metabolic markers measured. However, after women were separated according to menopausal status to further elucidate the potential effects of sex hormones on these outcomes, post-menopausal women exhibited a significant correlation between YFAS symptoms and TG after correcting for age, physical activity, total body fat percent and calories. While there are currently no past studies investigating the association between YFAS symptoms and metabolic abnormalities in women, other studies on disordered eating patterns such as binge eating have been found to have signification associations with T2D (62). The role of addictive food tendencies in T2D have also been found to be harmful. Varying levels of food addiction based on YFAS symptom counts were associated with significant increased in BMI among T2D patients in a sample that was 67.4% women, however, there was no adjustment for menopausal status (63). The limited associations seen in our female population may be due to the protective effects of lower body fat common among women which may help keep blood glucose and lipid profiles within a normal range in spite of problematic eating behaviors (64–66). However, this effect could be somewhat diminished in post-menopausal women as decreasing estrogen levels have been shown influence central fat accumulation and TG levels (67, 68)

The greatest strength of our study is the large number of individuals successfully recruited from the general population in conjunction with good screening efforts to control for potential confounders such as health disorders that could influence an individual's dietary habits or result in them presenting with addictive behaviors beyond that which is seen a general population. We have also benefitted from the ability to collect blood samples from every participant for reliable measurement of hormones and lipid profiles. Our extensive list of questionnaires to collect details on physical activity and dietary history add strength to the validity of our results as they allowed us to effectively control for these confounders. The use of DXA scanning to measure total body fat % ensured this particular confounding variable was measured using one of the most accurate methods available (69). One possible limitation present in our study could be that caloric intake and physical activity levels were self-reported, both of which were controlled for in this study. Self-reporting is of course prone to the self-reporting bias which researchers must be cognizant of. The number of food addicts used in the comparative analysis was also small as food addiction is only present in 5.4% of this particular population (70). It should also be noted that the exclusion of previously diagnosed food addicted individuals contributed to the modest correlation coefficients produced from the partial correlation analysis. However, exclusion of these outliers allowed us to better capture the true contribution of addictive eating tendencies to metabolic disturbance in a general population. Replications of this study would greatly benefit from a larger number of diagnosed food addicts. It is also possible that with a larger overall sample size, the correlation between YFAS symptoms and HOMA-IR in men may reach the required cut-off for statistical significance as it was narrowly missed in this analysis (Table 3). Future studies could also benefit from measuring a larger number of metabolic markers or health outcomes and their association with an addictive tendency toward food. Finally, an intervention study measuring metabolic characteristics in food addicts before and after treatment to address addictive eating behaviors would better highlight the impact these behaviors have on health and whether meaningful improvements in IR and lipid profiles can be achieved through this.

In conclusion, we have demonstrated that an addictive tendency toward food is associated with several metabolic characteristics that may contribute to insulin resistance and CVD in men of the general Newfoundland population and to a lesser extent, post-menopausal women. Our findings help elucidate the role of addictive food tendencies in metabolic disturbance in a general population and how this may impact men and women differently. Addressing these behaviors through therapy or lifestyle change may be beneficial to an individual's overall health. Furthermore, efforts to control addictive eating behaviors could potentially become a factor in preventative efforts concerning diabetes and CVD in the future.

## Data availability statement

The datasets ANALYZED for this study can be found in the Figshare repository [https://figshare.com/articles/Public_FA_Data_xlsx/6726065].

## Ethics statement

This study was approved by the Health Research Ethics Authority (HREA), Memorial University of Newfoundland, St. John's, Canada with project identification code 10.33. Written consent was provided by all who participated.

## Author contributions

MN analyzed data and wrote the manuscript. FC assisted with data analysis and participant recruitment. WG, GZ, WT, and ZS assisted in revision of manuscript. HZ conducted data collection and entry. GS designed the research and was responsible for the final content of the manuscript as Principal Investigator.

### Conflict of interest statement

The authors declare that the research was conducted in the absence of any commercial or financial relationships that could be construed as a potential conflict of interest.
